# Efficacy of hyperbaric oxygen therapy in treating sudden sensorineural hearing loss: an umbrella review

**DOI:** 10.3389/fneur.2024.1453055

**Published:** 2024-08-13

**Authors:** Xinghong Liu, Xianpeng Xu, Qiulian Lei, Xiaohua Jin, Xinxing Deng, Hui Xie

**Affiliations:** ^1^Department of Otorhinolaryngology, Chengdu University of Traditional Chinese Medicine, Chengdu, China; ^2^Department of Otorhinolaryngology, Hospital of Chengdu University of Traditional Chinese Medicine, Chengdu, China

**Keywords:** sudden sensorineural hearing loss, hyperbaric oxygen, efficacy, systematic review, meta-analysis

## Abstract

**Introduction:**

Our objective was to explore the efficacy of hyperbaric oxygen in the treatment of sudden sensorineural hearing loss by conducting an umbrella review of all existing evidence.

**Methods:**

We conducted an umbrella review, searching for related articles in the PubMed, Web of Science, Embase, and Scopus databases. The search period covered from the inception of each database until April 2024. We extracted authors, country of publication, time of publication, number of included studies and participants, interventions, summary of results, *P*-values, *I*^2^, relative risk (95% CI), and outcome measures. The methodological quality, evidence quality, and overlap rate of the included articles were assessed using AMSTAR 2, GRADE, and OVErviews (GROOVE).

**Results:**

Methodological quality was assessed using AMSTAR 2. Of the nine included articles, two were assessed as “high,” three as “moderate,” two as “low,” and the remaining two as “very low.” The quality of evidence was assessed using the GRADE system. It was found that the quality of evidence in most of the studies was unsatisfactory. It was found that there was a slight overlap among the included articles. Six studies reported positive results (OR 1.37; 95% CI, 1.17–1.61; *P* = 0.04), with high heterogeneity observed (*I*^2^ = 63%). Egger's test indicated bias (*P* = 0.000101). Three studies reported negative results (MD 1.49; 95% CI, −0.32 to 3.29; *P* = 0.43; *I*^2^ = 0%), with no significant bias detected (*P* = 0.106) according to Egger's test.

**Conclusion:**

HBO therapy is shown to be an effective treatment for SSNHL with fewer side effects. However, the methodological quality and evidence of the systematic reviews and meta-analysis included in this study were generally low. Therefore, more high-quality, large-scale, multi-center randomized controlled trials are needed in the future to verify the efficacy of HBO therapy for SSNHL.

**Systematic review registration:**

https://www.crd.york.ac.uk/prospero, identifier [CRD42024523651].

## 1 Introduction

Sudden sensorineural hearing loss (SSNHL) is defined as a rapid and unexplained hearing loss of at least 30 decibels (dB) across three contiguous frequencies, occurring within a 72-h period (1). The disease is more likely to occur unilaterally and may be accompanied by symptoms such as tinnitus, dizziness, ear discomfort, nausea and vomiting (2). Statistically, ~66,000 individuals are affected by SSNHL annually in the United States (3). The incidence of SSNHL is on the rise, potentially due to changes in work and lifestyle patterns (4). Given its sudden onset and severe symptoms, timely treatment is essential to prevent significant impacts on a patient's health and quality of life (5). Early and effective diagnosis and treatment are therefore critical for SSNHL patients.

The etiology of SSNHL is not fully understood, but it is believed to be associated with factors such as viral infections, vascular and endothelial abnormalities, immune-mediated mechanisms, endolymphatic hydrops, and psychosomatic factors (6–8). There are multiple treatment options available for this disease, yet there is still no standardized treatment plan in place (9). Clinical interventions often include systemic or local administration of steroids, diuretics, antiviral drugs, and vasodilators, all aimed at increasing blood flow and oxygen concentration in the inner ear (10–12). Additionally, hyperbaric oxygen (HBO) therapy is a common salvage treatment (13). This therapy involves the controlled intake of pure oxygen to enhance oxygen tension in the cochlea and promote tissue repair, thereby improving hearing outcomes in patients (14).

Although numerous studies have investigated the use of HBO therapy for SSNHL, some have confirmed its positive impact on patients' hearing, yet the efficacy of HBO compared to other treatments remains highly controversial (15–18). We have considered the discrepancies in existing systematic reviews and meta-analyses regarding the efficacy of HBO in treating SSNHL. Therefore, this study employed an umbrella review to compile all previous evidence and comprehensively evaluate the efficacy of HBO in treating SSNHL, as well as to assess the validity of the existing evidence. Our goal is to provide a comprehensive and objective summary of evidence-based medicine for the treatment of this condition.

## 2 Methods and materials

### 2.1 Protocol and registration

This study followed the Preferred Reporting Items for Systematic Reviews and Meta-analysis (PRISMA) (19). This study is registered in PROSPERO (CRD42024523651).

### 2.2. Search strategy

To investigate the efficacy of HBO therapy for SSNHL, two researchers (XHL and XPX) independently searched for related articles in the PubMed, Web of Science, Embase, and Scopus databases. The search period covered from the inception of each database until March 31, 2024. Search terms: “sudden deafness,” “Sudden Hearing Loss,” “Deafness, Sudden,” “Sudden Deafness,” “sudden sensorineural hearing loss,” “Hyperbaric Oxygenation,” “Hyperbaric Oxygen Therapy^*^,” “Oxygen Therapy^*^, Hyperbaric,” “Therapy^*^, Hyperbaric Oxygen,” “Oxygenations, Hyperbaric,” “Hyperbaric Oxygen,” “Meta-Analy^*^,” “Meta-Analysis as Topic,” “Systematic Review^*^,” “Systematic Reviews as Topic.” In addition, a reference review of relevant studies and a search of the gray literature were conducted to avoid missing relevant articles in the initial search. We have listed the detailed search strategies for each database in the Supplementary Appendix (p. 5).

### 2.3. Selection of studies

Systematic reviews and meta-analyses that assessed the efficacy of HBO therapy in the treatment of SSNHL were included. Each study was independently evaluated by two investigators (XHL and XPX). The titles and abstracts of the articles were screened by the two researchers independently to determine their relevance to the topic. In cases where there was a disagreement that could not be resolved through discussion, a third researcher (HX) made the final decision on whether to include the article. Articles that were excluded based on full-text screening, along with the reasons for their exclusion, are listed in the Supplementary Appendix (p. 6).

### 2.4 Data extraction

After finalizing the data extraction table, two researchers (XHJ and XXD) independently extracted the following data from each systematic review and meta-analysis: authors, country of publication, time of publication, number of included studies and participants, interventions, summary of results, *P*-values, *I*^2^, relative risk (95% CI), and outcome measures. All data were extracted independently by both researchers.

### 2.5 Methodological and evidence quality evaluation

Two researchers (XHL and XPX) independently assessed each included systematic review and meta-analysis. The quality of the methodology was evaluated using AMSTAR 2, a 16-item tool that measures consistency, reliability, and feasibility, with each item categorized as “yes,” “partially yes,” or “no” (20). The quality of evidence was assessed using the GRADE system, which clearly defines the quality of evidence and the strength of recommendations, categorizing evidence as “high,” “medium,” “low,” “very low,” or “unable to make a recommendation” (21). The overlap of major studies included in the literature can potentially mislead results. To measure this overlap, we used the OVErviews (GROOVE) tool, which calculates evidence matrices and corrected coverage area (CCA). The overlap is categorized as slight if CCA < 5%, moderate if CCA ≥5% and < 10%, high if CCA ≥10% and < 15%, and very high if CCA ≥15% (22).

### 2.6 Statistical analysis

We extracted data on the efficacy of HBO therapy in the treatment of SSNHL. The 95% CI reported for each study was used to assess overall efficacy. Heterogeneity among studies was evaluated using the *I*^2^, with values > 50% indicating high heterogeneity. Publication bias in systematic reviews and meta-analyses was assessed using Egger's test, with a *P*-value < 0.1 suggesting the presence of bias (23).

## 3 Results

### 3.1 Search results

**Figure 1:**
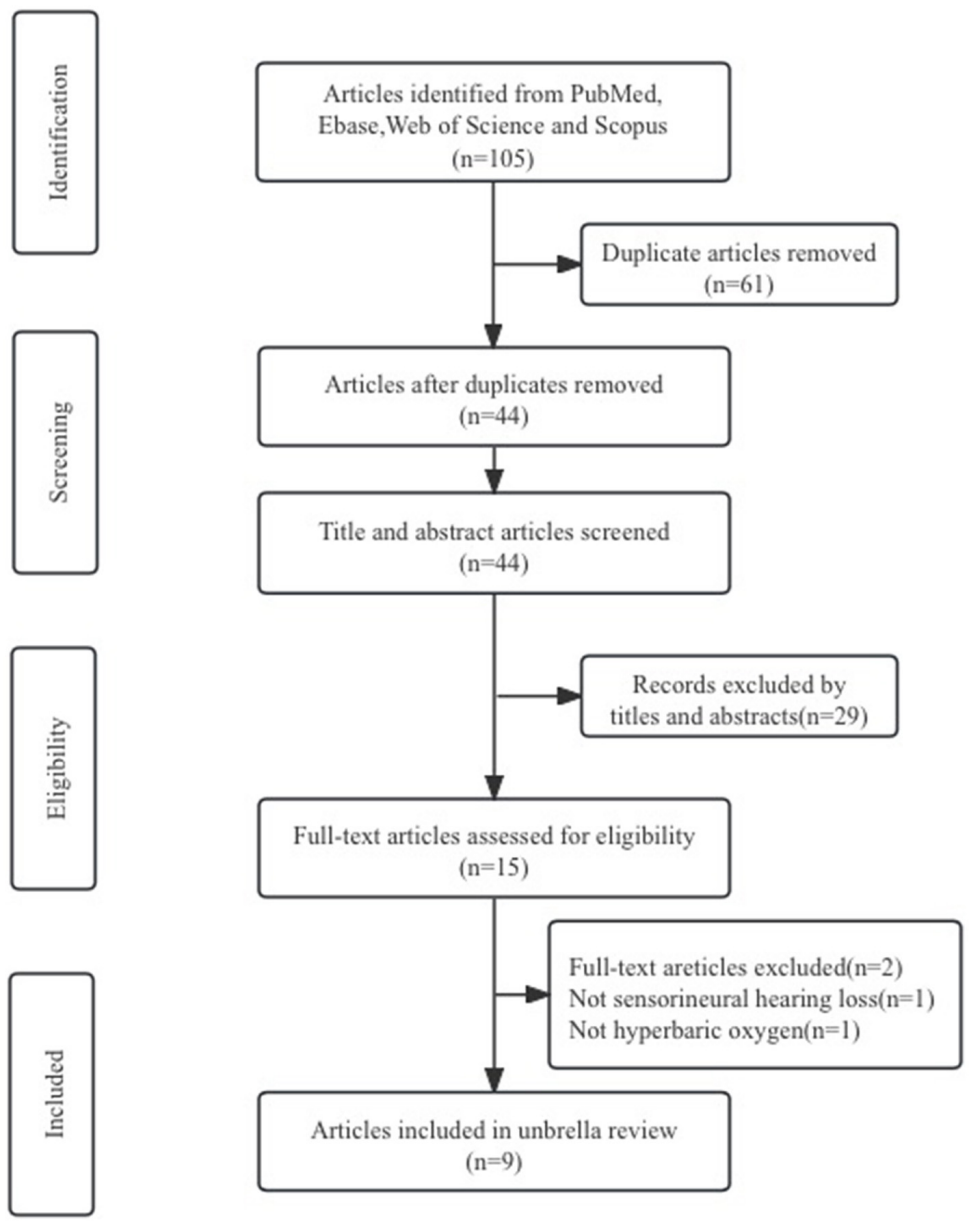
Flow chart of the literature selection.

Based on the established search strategy, we initially retrieved 105 articles. After removing 61 duplicate articles, 33 articles were excluded after reading the titles and abstracts as they were irrelevant to the selected topic. After reading the full text, two more articles were excluded. Ultimately, nine systematic reviews and meta-analyses were included in this umbrella review through the literature screening process, as shown in Figure 1. We summarized the efficacy indicators of HBO treatment for SSNHL, using hearing gain (HG) or pure-tone audiometric (PTA) as references for evaluating the efficacy of HBO treatment for SSNHL, and extracted this data from the included studies. The effectiveness results of HBO treatment for SSNHL are presented in Table 1.

**Table 1:** Characteristic of the included studies.

**References**	**Country **	**No. of studies (participants) **	**Treatmentintervention **	**Controlintervention **	**Outcomes **	**Results summary **	***P*-value **	***I* ^2^**	**Effect size **	**95 % CI **	**AMSTAR 2 **	**Qualityassessment **
Kuo et al. (24)	Taiwan	4 (121)	HBO	ITS	HG	There were no significant differences between the HBO and ITS groups	< 0.05	0%	MD	2.70 (−0.63 to 6.02)	Moderate	Cochrane
Conlin and Parnes (25)	Canada	1 (34)	HBO + MT	MT	PTA	Hearing improvement was higher in the HBO group	< 0.05	NR	NR	NR	Critically low	The Users' Guides to the Medi-calLiterature
Eryigit et al. (26)	Netherla-nds	16 (1,361)	HBO + steroid	steroid	HG	HBO may be beneficial for patients with severe to very severe hearing Loss	0.005	NR	NR	NR	Low	RoBANS
Bennett et al. (27)	Australia	6 (304)	HBO	MT	PTA	HBO improves hearing and does not justify routine use.	0.02	0%	RR	1.39 (1.05 to 1.84)	low	Cochrane
Rhee et al. (28)	South Korea	19 (2,401)	HBO + MT	MT	PTA	Addition of HBO to standard MT is a reasonable treatment option for SSNHL patients	< 0.001	79.3%	OR	1.43 (1.20 to 1.67)	High	Cochrane
Lei et al. (29)	China	6 (165)	HBO	ITS	PTA	No significant difference in outcome between HBO and ITS treatments	0.51	0%	MD	0.55 (−1.76 to 2.86)	Moderate	Jadad
Joshua et al. (30)	Canada	3 (150)	HBO + MT	MT	PTA	HBO as part of a combination treatment was significantly associated with improved hearing outcomes in patients with SSNHL	0.004	0%	OR	4.32 (1.60 to 11.68)	High	Cochrane
Lin et al. (31)	Taiwan	20 (125)	HBO	PSI, ITS, SS	PTA	There is no convincing evidence that HBO is beneficial in the treatment of SSNHL	0.4649	76%	MD	3.65 (−2.08 to 9.38)	Moderate	Cochrane
Ma et al. (32)	China	39 (2,067)	HBO + MT	MT	HG	HBO combined with medication is more effective in treating SSNHL	< 0.00001	50%	RR	1.26 (1.22 to 1.30)	Critically low	Cochrane

HBO, hyperbaric oxygen; ITS, intratympanic steroids; HG, hearing gain; MD, mean difference; MT, medical therapy; PTA, pure tone averages; NR, not reported; RR, relative risk; PSI, postauricular steroid injection; SS, systemic steroid; OR, odds ratio.

### 3.2 Characteristics of included studies

The nine systematic reviews and meta-analyses included in this study were all published between 2005 and 2024. These papers analyzed a total of 114 studies (randomized controlled trials or non-randomized controlled trials) involving 6,728 patients. There were slight differences in the interventions used in the experimental groups: four articles treated with HBO, four articles treated with a combination of oral routine medication and HBO, and one article treated with a combination of HBO and systemic steroids (SS). The control group interventions also varied, with five articles based on oral routine medication and four articles based on systemic or topical steroid use. Six of the included papers assessed the quality of the original literature using Cochrane criteria, one used the Jadad scale, one used RoBANS, and one used the Users' Guides to the Medical Literature. The main characteristics of the included papers are detailed in Table 1.

### 3.3 Quality evaluation

Methodological quality was assessed using AMSTAR 2. Of the nine included articles, two were assessed as “high,” three as “moderate,” two as “low,” and the remaining two as “very low.” Most articles did not provide a list of excluded literature or state the reasons for exclusion. Some articles interpreted or discussed the results without considering the risk of bias in the included studies. Additionally, in some articles, the researchers did not provide a reasonable explanation or discussion of the heterogeneity of the findings. Details of the specific assessments are shown in Table 2.

**Table 2:** Results of the AMSTAR 2 assessment.

**References**	**Q1**	**Q2**	**Q3**	**Q4**	**Q5**	**Q6**	**Q7**	**Q8**	**Q9**	**Q10**	**Q11**	**Q12**	**Q13**	**Q14**	**Q15**	**Q16**	**Overall quality**
Kuo et al. (24)	Y	PY	Y	PY	Y	Y	PY	Y	Y	Y	Y	Y	Y	N	Y	Y	M
Conlin and Parnes (25)	Y	PY	Y	PY	N	N	N	Y	Y	Y	N	Y	N	N	N	Y	CL
Eryigit et al. (26)	Y	PY	Y	PY	Y	Y	PY	Y	Y	Y	Y	Y	Y	N	N	Y	L
Bennett et al. (27)	Y	PY	Y	PY	Y	Y	N	Y	Y	Y	Y	Y	Y	Y	Y	Y	L
Rhee et al. (28)	Y	PY	Y	PY	Y	Y	PY	Y	Y	Y	Y	Y	Y	Y	Y	Y	H
Lei et al. (29)	Y	PY	Y	PY	Y	Y	PY	Y	Y	Y	Y	Y	Y	N	Y	Y	M
Joshua et al. (30)	Y	PY	Y	Y	Y	Y	PY	Y	Y	Y	Y	Y	Y	Y	Y	Y	H
Lin et al. (31)	Y	Y	Y	PY	Y	Y	Y	Y	Y	Y	Y	Y	Y	N	Y	Y	M
Ma et al. (32)	Y	PY	Y	PY	N	Y	N	N	Y	Y	Y	Y	N	Y	N	Y	CL

Y, yes; PY, partial yes; N, no; CL, critically low; L, low; M, moderate; H, high.

The quality of evidence was assessed using the GRADE system. It was found that the quality of evidence in most of the studies was unsatisfactory. Among the included articles, there was only one with high-quality evidence, one with moderate-quality evidence, six with low-quality evidence, and 1 with very low-quality evidence. Inconsistency was the most common downgrading factor among all programs, likely due to differences in the interventions in the included studies. The second most common downgrading factors were risk of bias, accuracy, and limitations. No items were downgraded for indirectness. Details of the specific assessments are shown in Table 3.

**Table 3:** Assessments of the GRADE.

**References**	**Risk of bias**	**Inconsistency**	**Indirectness**	**Imprecision**	**Publication bias**	**Evidence quality**
Kuo et al. (24)	0	−1	0	−1	0	Low
Conlin and Parnes (25)	0	−1	0	−1	−1	Very Low
Eryigit et al. (26)	0	−1	0	0	−1	Low
Bennett et al. (27)	−1	0	0	0	−1	Low
Rhee et al. (28)	0	0	0	0	0	High
Lei et al. (29)	−1	−1	0	0	0	Low
Joshua et al. (30)	0	0	0	0	−1	Moderate
Lin et al. (31)	0	−1	0	−1	0	Low
Ma et al. (32)	0	−1	0	0	−1	Low

VL, very low; L, low; M, moderate; H, high.

**Figure 2:**
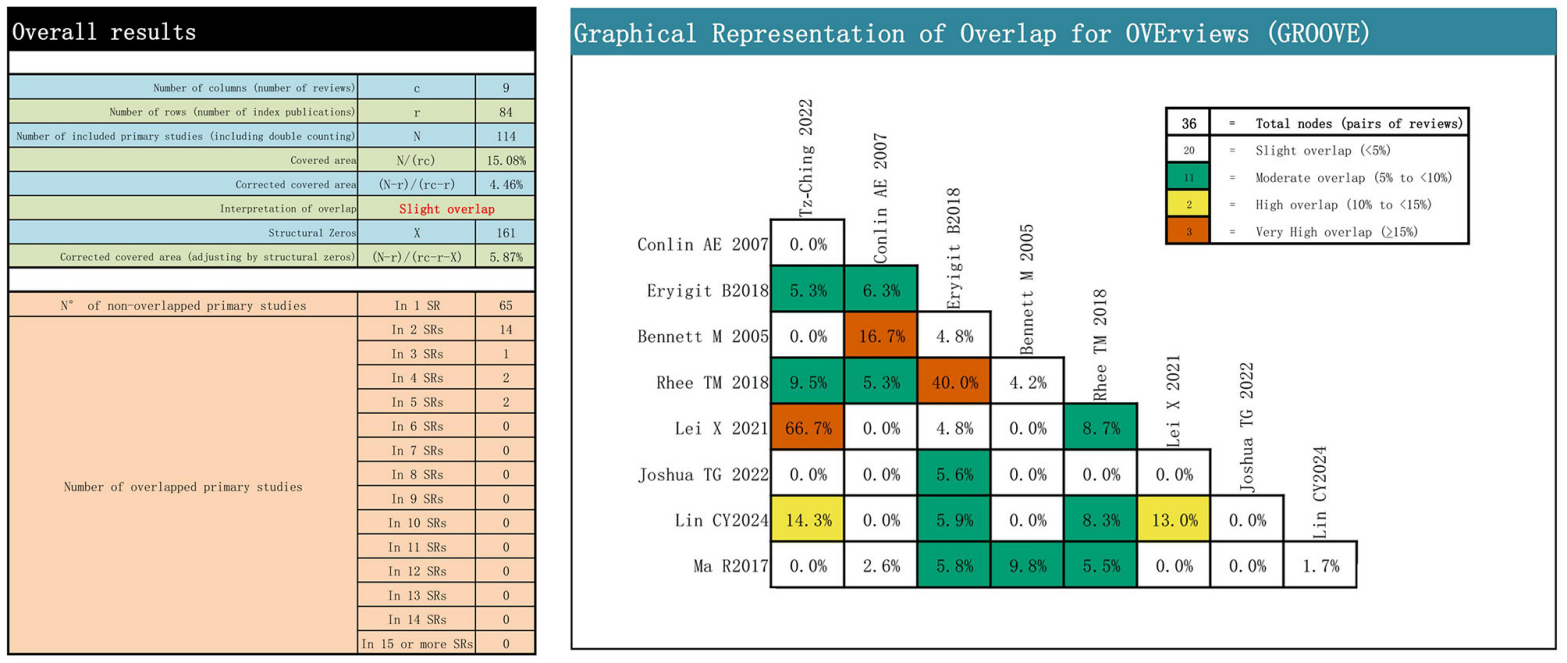
Overlapping of the included reviews.

The overlap of primary studies in the included literature was assessed using the GROOVE tool. It was found that there was a slight overlap among the included articles. The tool uses the formula (N-r)/(rc-r) to calculate the overlap rate. There are a total of 36 nodes between the included articles, of which 20 are slightly overlapping, 11 are moderately overlapping, two are highly overlapping, and three are very highly overlapping. The overall overlap was mild, at 4.46%. Detailed assessment results are shown in Figure 2.

### 3.4. Results of the effectiveness of HBO in treating SSNHL

**Figure 3:**
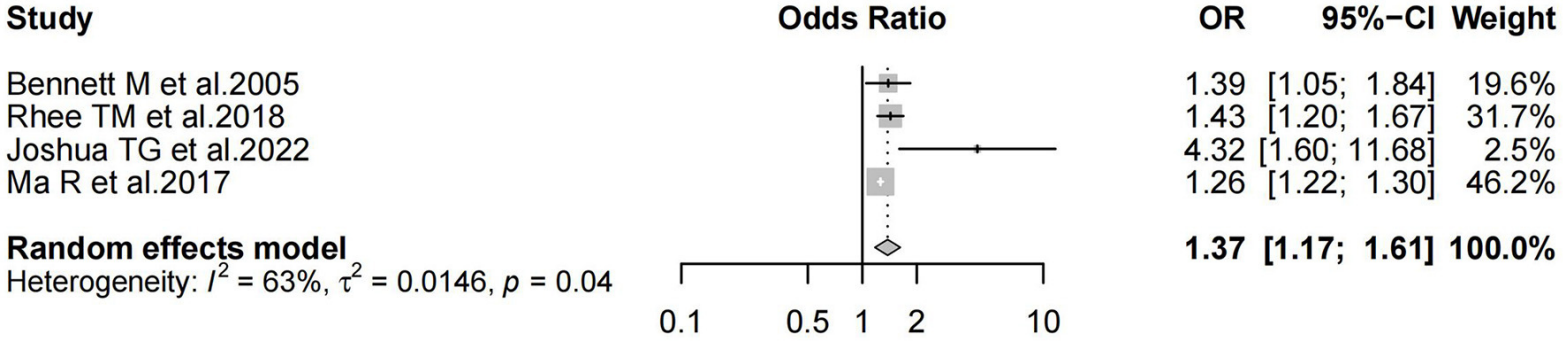
Forest plot of Odds Ratio for positive results.

**Figure 4:**
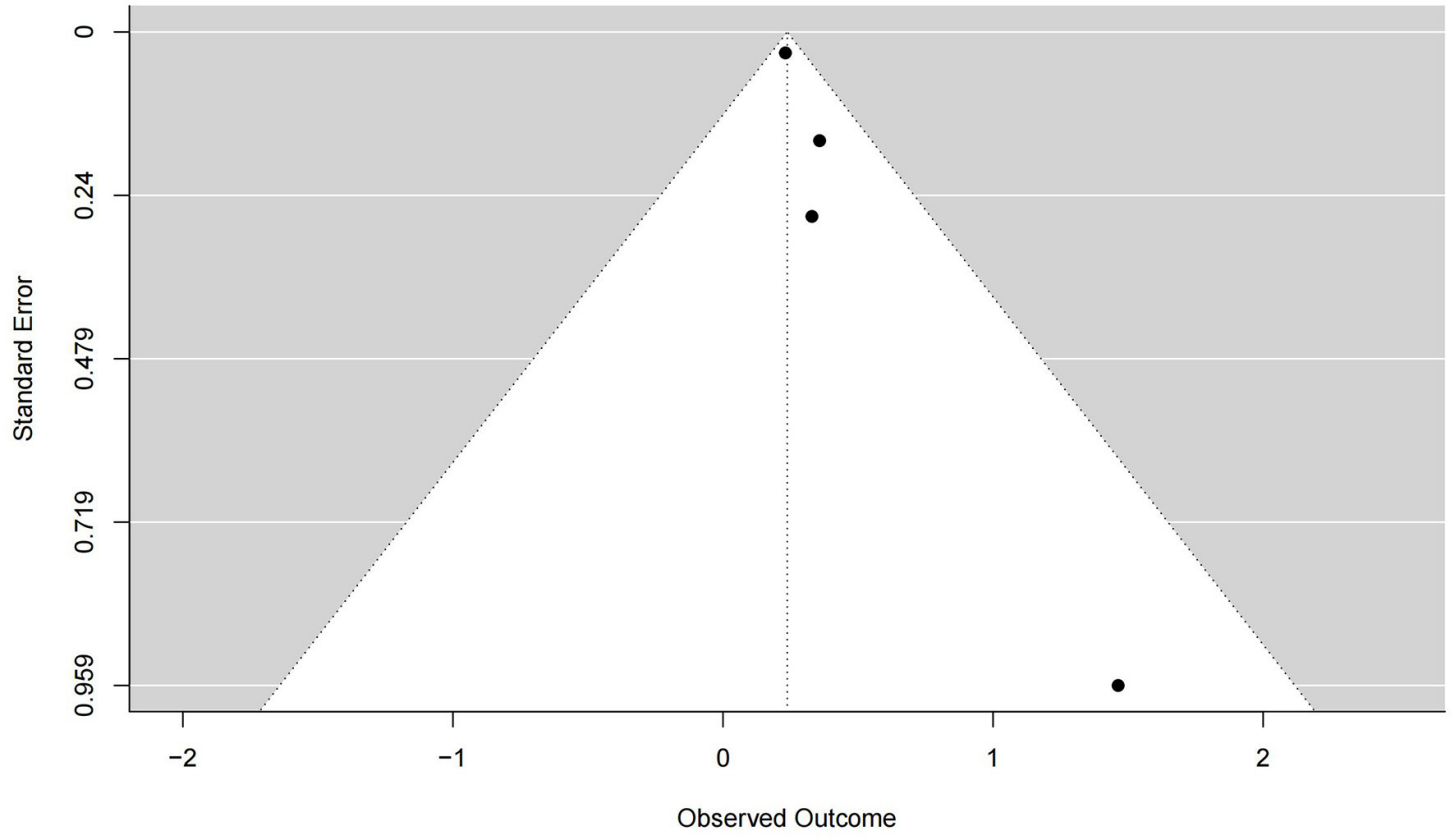
Funnel plot with the Egger test of positive results.

Six studies reported positive results, and the positive results indicated that the HBO experimental group was more efficacious than the control group. Of the positive results, two studies resulted only in outcomes without reporting 95% CI. Among them, Conlin and Parnes (25) concluded that HBO with oral medication is more efficacious than oral medication only; Eryigit et al. (26) concluded that HBO with steroid was superior in efficacy to steroid only; The remaining four studies both concluded and also reported 95% CI. Among them, Bennett et al. (27) reported superior efficacy of HBO treatment over oral drug therapy (RR 1.39; 95% CI, 1.05–1.84); Rhee et al. (28) reported that HBO with oral medication was more efficacious than treatment with oral medication only (OR 1.43; 95% CI, 1.20–1.67); Joshua et al. (30) reported better efficacy of HBO with oral medication than oral medication only (OR 4.32; 95% CI, 1.60–11.68); Ma et al. (32) reported better efficacy of HBO addition to medication than oral medication only (RR 1.26; 95% CI, 1.22–1.30). We extracted the 95% CI reported for each study to assess overall efficacy (OR 1.37; 95% CI, 1.17–1.61; *P* = 0.04; Figure 3). The results of our study showed high heterogeneity (*I*^2^ = 63%). The funnel plot (Figure 4) as well as the Egger's test showed (*P* = 0.000101) that the study was biased.

**Figure 5:**
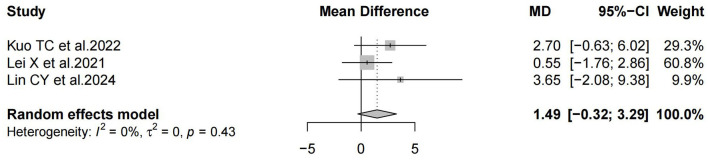
Forest plot of Odds Ratio for negative results.

**Figure 6:**
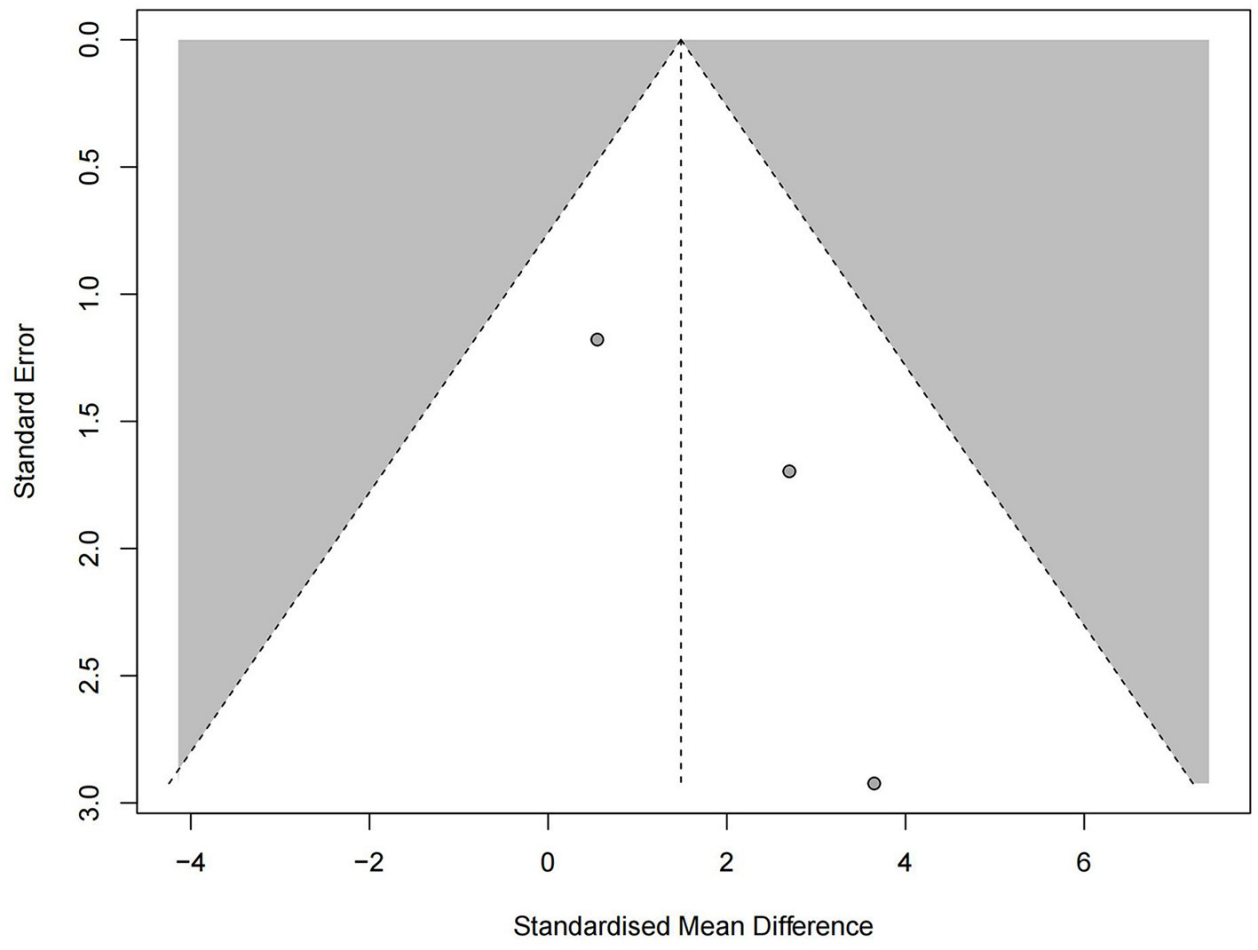
Funnel plot with the Egger test of negative results.

Three studies reported negative results, and the negative results indicated that the HBO treatment group was not significantly different from the control group. All three studies reported the 95% CI. Among them, Kuo et al. (24) reported no significant difference in the effect of HBO treatment vs. intratympanic steroids (ITS) treatment (MD 2.70; 95% CI, −0.63 to 6.02); Lei et al. (29) reported that there was no significant difference in the effect of HBO treatment vs. ITS treatment (MD 0.55; 95% CI, −1.76 to 2.86); Lin et al. (31) showed that HBO therapy has no significant additional benefits compared to PSI and ITS. For patients diagnosed with refractory sudden sensorineural hearing loss (PTA improvement < 10 dB after initial systemic steroid therapy), HBO therapy alone may be useful. However, due to the lack of multi-group RCTs with sufficiently large sample sizes, there is no convincing evidence that HBO is beneficial for the treatment of SSNHL (MD 3.65; 95% CI, −2.08 to 9.38). We extracted the 95% CI reported from each study to assess overall efficacy (MD 1.49; 95% CI, −0.32 to 3.29; *P* = 0.43; *I*^2^ = 0%) (Figure 5). The funnel plot (Figure 6) and Egger's test showed (*P* = 0.106) no significant bias.

## 4 Discussion

HBO has served as a treatment for SSNHL for over four decades (33). In recent years, there has been considerable controversy regarding the treatment of SSNHL with HBO (34, 35). The number of systematic reviews and meta-analyses of HBO for the treatment of SSNHL is relatively small. Unfortunately, discrepancies in evaluation methodologies have resulted in inconsistent and less effective quality of assessments.

To address these issues, we conducted a detailed summary of nine studies to further analyze the methodological quality, evidence, and reporting standards of HBO for SSNHL treatment. Among these studies, six reported HBO's effectiveness, while three found no significant difference between HBO and alternative therapies. HBO, along with ITS, is recognized as a salvage therapy for SSNHL (36, 37). Conlin and Parnes (25) concluded that HBO combined with standard medication yielded a higher rate of improvement in PTA compared to standard medication alone, albeit based on limited methodological quality. The principle behind HBO therapy for SSNHL is to dilate the blood vessels in the Corti organ and other inner ear organs, thereby combating vascular damage and oxidative stress (38). Eryigit et al. (26) demonstrated that HBO with steroids had superior efficacy over steroids alone. However, HBO therapy also has some limitations, such as treatment time dependence, the later the treatment time, the worse the effect, and higher the cost of HBO, which will bring greater economic burden to patients (39–41). Nevertheless, HBO remains a relatively safe treatment option, with minimal adverse effects and only minor complications (42). Bennett et al. (27) reported a 25% improvement in patients' mean hearing, yet due to the small number of included studies and poor quality, they refrained from justifying routine HBO use for SSNHL. Rhee et al. (28) showed that HBO combined with standard medication was more effective in improving hearing compared to medication alone, especially in female patients and those with severe hearing loss at baseline. Joshua et al. (30) found HBO with medication to be more efficacious than medication alone, with efficacy potentially related to treatment duration (>1,200 min). HBO therapy effectively reduces endolymphatic hydrops due to bacterial and viral infections, thereby improving hearing in SSNHL patients (43–45). Early combined HBO treatment may also lead to better hearing recovery (46, 47).

Some studies, however, have reported no significant difference between HBO and other therapies for SSNHL. For instance, Kuo et al. (24) concluded that HBO and ITS as salvage therapies exhibited no significant difference in mean hearing gains, although salvage therapy outperformed no treatment. Studies have shown that HBO is used to deliver increased oxygen to the inner ear in a high-pressure environment to improve patient hearing (48). While HBO delivers increased oxygen to the inner ear, its application is limited to specific devices, preventing its inclusion in standard SSNHL treatment protocols (1). Additionally, some studies suggest a partial discomfort syndrome associated with HBO treatment (49, 50). For example, a study by Lei et al. (29) suggests that HBO therapy may cause discomfort such as otitis media or ear fullness in patients, and its efficacy does not show significant differences compared to ITS treatment. Similarly, Lin et al. (31) observed no significant advantage of HBO therapy and reported the possibility of uncomfortable symptoms such as middle ear barotrauma and claustrophobia.

The primary advantage of this study lies in its use of an umbrella review to re-evaluate existing evidence and synthesize higher-level evidence. This has certain implications for clinicians in deciding whether to choose HBO therapy for SSNHL. However, the study has several limitations: (1) According to the AMSTAR 2 methodology, only two of the included studies were categorized as high-quality studies because most systematic reviews and meta-analyses did not consider the risk of bias in the included literature and did not account for the heterogeneity of the study results; (2) The combination of other medications during HBO therapy may also affect the outcome. In addition, the number of included studies and patients was small in this study, which may also affect our conclusions. In view of the shortcomings of this study, further high-quality studies are therefore needed.

## 5 Conclusion

In conclusion, HBO emerges as an active and effective treatment for SSNHL, boasting fewer adverse effects compared to alternative therapies, despite its notable drawbacks such as the high cost of treatment. However, the methodological quality and evidence of the systematic reviews and meta-analyses included in this study are generally low, so this result must be considered with caution. Therefore, the imperative for future research is clear. More high-quality, large-scale, multicenter, randomized clinical controlled trials are essential to robustly validate the efficacy of HBO in SSNHL treatment. These endeavors are pivotal for advancing our understanding and ensuring the validity of HBO as a therapeutic modality for SSNHL.

## Data availability statement

The original contributions presented in the study are included in the article/Supplementary material, further inquiries can be directed to the corresponding author.

## Author contributions

XL: Writing – original draft, Writing – review & editing. XX: Writing – original draft, Writing – review & editing. QL: Writing – original draft, Writing – review & editing. XJ: Writing – original draft, Writing – review & editing. XD: Writing – original draft, Writing – review & editing. HX: Writing – original draft, Writing – review & editing.
