# Supplementary material for: Efficacy of hyperbaric oxygen therapy in treating sudden sensorineural hearing loss: an umbrella review
Source: Front Neurol. 2024 Aug 13;15:1453055. doi: 10.3389/fneur.2024.1453055 (PMC11347443; doi:10.3389/fneur.2024.1453055)
Supplement: Supplementary file 1 [file Table_1.docx]

***Supplementary material***

**Efficacy of hyperbaric oxygen therapy in treating sudden sensorineural hearing loss:**

**an umbrella review**

1. **PRISMA checklist**

| Section and Topic | Item # | Checklist item | Location where item is reported |
| --- | --- | --- | --- |
| TITLE | | |  |
| Title | 1 | Identify the report as a systematic review. | Manuscript |
| ABSTRACT | | |  |
| Abstract | 2 | See the PRISMA 2020 for Abstract checklist. | Supplementary material |
| INTRODUCTION | | |  |
| Rationale | 3 | Describe the rationale for the review in the context of existing knowledge. | Manuscript |
| Objectives | 4 | Provide an explicit statement of the objective(s) or question(s) the review addresses. | Manuscript |
| METHODS | | |  |
| Eligibility criteria | 5 | Specify the inclusion and exclusion criteria for the review and how studies were grouped for the syntheses. | Manuscript |
| Information sources | 6 | Specify all databases, registers, websites, organisations, reference lists and other  sources searched or consulted to identify studies. Specify the date when each source was last searched or consulted. | Manuscript Figure 1 |
| Search strategy | 7 | Present the full search strategies for all databases, registers and websites, including any filters and limits used. | Manuscript Supplementary  material |
| Selection process | 8 | Specify the methods used to decide whether a study met the inclusion criteria of the  review, including how many reviewers screened each record and each report retrieved, whether they worked independently, and if applicable, details of automation tools used in the process. | Manuscript |
| Data collection process | 9 | Specify the methods used to collect data from reports, including how many reviewers collected data from each report, whether they worked independently, any processes  for obtaining or confirming data from study investigators, and if applicable, details of automation tools used in the process. | Manuscript |
| Data items | 10a | List and define all outcomes for which data were sought. Specify whether all results that were compatible with each outcome domain in each study were sought (e.g. for all measures, time points, analyses), and if not, the methods used to decide which  results to collect. | Manuscript |
|  | 10b | List and define all other variables for which data were sought (e.g. participant and  intervention characteristics, funding sources). Describe any assumptions made about any missing or unclear information. | Manuscript |
| Study risk of bias assessment | 11 | Specify the methods used to assess risk of bias in the included studies, including  details of the tool(s) used, how many reviewers assessed each study and whether they worked independently, and if applicable, details of automation tools used in the  process. | Manuscript |
| Effect measures | 12 | Specify for each outcome the effect measure(s) (e.g. risk ratio, mean difference) used in the synthesis or presentation of results. | Manuscript |
| Synthesis methods | 13a | Describe the processes used to decide which studies were eligible for each synthesis (e.g. tabulating the study intervention characteristics and comparing against the  planned groups for each synthesis (item #5)). | Manuscript |
|  | 13b | Describe any methods required to prepare the data for presentation or synthesis, such as handling of missing summary statistics, or data conversions. | Manuscript |
|  | 13c | Describe any methods used to tabulate or visually display results of individual studies and syntheses. | Manuscript |
|  | 13d | Describe any methods used to synthesize results and provide a rationale for the  choice(s). If meta-analysis was performed, describe the model(s), method(s) to  identify the presence and extent of statistical heterogeneity, and software package(s) used. | Manuscript |
|  | 13e | Describe any methods used to explore possible causes of heterogeneity among study results (e.g. subgroup analysis, meta-regression). | Manuscript |
|  | 13f | Describe any sensitivity analyses conducted to assess robustness of the synthesized results. | Manuscript |
| Reporting bias assessment | 14 | Describe any methods used to assess risk of bias due to missing results in a synthesis (arising from reporting biases). | Manuscript |
| Certainty | 15 | Describe any methods used to assess certainty (or confidence) in the body of | Manuscript |

| Section and Topic | Item # | Checklist item | Location where item is reported |
| --- | --- | --- | --- |
| assessment |  | evidence for an outcome. |  |
| RESULTS | | |  |
| Study selection | 16a | Describe the results of the search and selection process, from the number of records identified in the search to the number of studies included in the review, ideally using a flow diagram. | Manuscript  Figure 1 |
|  | 16b | Cite studies that might appear to meet the inclusion criteria, but which were excluded, and explain why they were excluded. | Supplementary material |
| Study  characteristics | 17 | Cite each included study and present its characteristics. | Manuscript  Table 1 |
| Risk of bias in studies | 18 | Present assessments of risk of bias for each included study. | Manuscript  Table 1 |
| Results of  individual studies | 19 | For all outcomes, present, for each study: (a) summary statistics for each group (where appropriate) and (b) an effect estimate and its precision (e.g.  confidence/credible interval), ideally using structured tables or plots. | Manuscript  Table 1 |
| Results of syntheses | 20a | For each synthesis, briefly summarise the characteristics and risk of bias among contributing studies. | Manuscript  Table 1 |
|  | 20b | Present results of all statistical syntheses conducted. If meta-analysis was done,  present for each the summary estimate and its precision (e.g. confidence/credible  interval) and measures of statistical heterogeneity. If comparing groups, describe the direction of the effect. | Manuscript  Figure 3-6 |
|  | 20c | Present results of all investigations of possible causes of heterogeneity among study results. | Manuscript  Table 2-3 |
|  | 20d | Present results of all sensitivity analyses conducted to assess the robustness of the synthesized results. | Manuscript  Table 1 |
| Reporting biases | 21 | Present assessments of risk of bias due to missing results (arising from reporting biases) for each synthesis assessed. | Manuscript |
| Certainty of evidence | 22 | Present assessments of certainty (or confidence) in the body of evidence for each outcome assessed. | Manuscript |
| DISCUSSION | | |  |
| Discussion | 23a | Provide a general interpretation of the results in the context of other evidence. | Manuscript |
|  | 23b | Discuss any limitations of the evidence included in the review. | Manuscript |
|  | 23c | Discuss any limitations of the review processes used. | Manuscript |
|  | 23d | Discuss implications of the results for practice, policy, and future research. | Manuscript |
| OTHER INFORMATION | | |  |
| Registration and protocol | 24a | Provide registration information for the review, including register name and registration number, or state that the review was not registered. | Manuscript |
|  | 24b | Indicate where the review protocol can be accessed, or state that a protocol was not prepared. | Manuscript |
|  | 24c | Describe and explain any amendments to information provided at registration or in the protocol. | No amendments to information |
| Support | 25 | Describe sources of financial or non-financial support for the review, and the role of the funders or sponsors in the review. | Manuscript |
| Competing interests | 26 | Declare any competing interests of review authors. | Manuscript |
| Availability of data, code and other  materials | 27 | Report which of the following are publicly available and where they can be found:  template data collection forms; data extracted from included studies; data used for all analyses; analytic code; any other materials used in the review. | Manuscript |

*From:* Page MJ, McKenzie JE, Bossuyt PM, Boutron I, Hoffmann TC, Mulrow CD, et al. The PRISMA 2020 statement: an updated guideline for reporting systematic reviews. BMJ 2021;372:n71. doi: 10.1136/bmj.n71

1. **PRISMA Abstract checklist**

Some checklist items cannot be included in the abstract due to the word count restriction. (<170 words)

| Section and Topic | Item # | Checklist item | Reported (Yes/No) |
| --- | --- | --- | --- |
| TITLE | | |  |
| Title | 1 | Identify the report as a systematic review. | Yes |
| BACKGROUND | | |  |
| Objectives | 2 | Provide an explicit statement of the main objective(s) or question(s) the review addresses. | Yes |
| METHODS | | |  |
| Eligibility criteria | 3 | Specify the inclusion and exclusion criteria for the review. | Yes |
| Information sources | 4 | Specify the information sources (e.g. databases, registers) used to identify studies and the date when each was last searched. | Yes |
| Risk of bias | 5 | Specify the methods used to assess risk of bias in the included studies. | Yes |
| Synthesis of results | 6 | Specify the methods used to present and synthesise results. | No |
| RESULTS | | |  |
| Included studies | 7 | Give the total number of included studies and participants and summarise relevant characteristics of studies. | Yes |
| Synthesis of results | 8 | Present results for main outcomes, preferably indicating the number of included studies and participants for each. If meta-analysis was done, report the summary estimate and  confidence/credible interval. If comparing groups, indicate the direction of the effect (i.e. which group is favoured). | Yes |
| DISCUSSION | | |  |
| Limitations of evidence | 9 | Provide a brief summary of the limitations of the evidence included in the review (e.g. study risk of bias, inconsistency and imprecision). | No |
| Interpretation | 10 | Provide a general interpretation of the results and important implications. | No |
| OTHER | | |  |
| Funding | 11 | Specify the primary source of funding for the review. | No |
| Registration | 12 | Provide the register name and registration number. | No |

From: Page MJ, McKenzie JE, Bossuyt PM, Boutron I, Hoffmann TC, Mulrow CD, et al. The PRISMA 2020 statement: an updated guideline for reporting systematic reviews. BMJ 2021;372:n71. doi: 10.1136/bmj.n71

|  |
| --- |

1. **Full search strategy**

| **Database** | **No** | **Search Query** | **Results** |
| --- | --- | --- | --- |
| **PubMed** | | | |
|  | #1 | ((((sudden deafness[MeSH Terms]) OR (Sudden Hearing Loss[Title/Abstract])) OR (Deafness,  Sudden[Title/Abstract])) OR (Sudden Deafness[Title/Abstract])) OR (sudden sensorineural hearing  loss[Title/Abstract]) | 4917 |
|  | #2 | ((((Hyperbaric Oxygenation[MeSH Terms]) OR (Hyperbaric Oxygen Therapy*[Title/Abstract]))  OR (Oxygen Therapy*, Hyperbaric[Title/Abstract])) OR (Therapy*, Hyperbaric Oxygen  [Title/Abstract])) OR (Oxygenations, Hyperbaric[Title/Abstract]) | 16423 |
|  | #3 | (((((Meta-Analysis as Topic[MeSH Terms]) OR (Meta-Analysis[Publication Type])) OR (Meta-  Analy*)) OR (Systematic Reviews as Topic[MeSH Terms])) OR (Systematic Review[Publication  Type])) OR (Systematic Review*) | 767894 |
|  | #4 | #1 AND #2 AND #3 | 27 |
| **Web of science** | | | |
|  | #1 | TS=(Sudden Hearing Loss OR Deafness, Sudden OR Sudden Deafness) | 2304 |
|  | #2 | TS=(Hyperbaric Oxygenation* OR Oxygenations, Hyperbaric OR Hyperbaric Oxygen Therapy*  OR Oxygen Therapy*, Hyperbaric OR Therapy*, Hyperbaric Oxygen) | 4806 |
|  | #3 | ALL=(Meta-Analysis as Topic OR Meta-Analysis OR Meta-Analy* OR Systematic Reviews as  Topic OR Systematic Review OR Systematic Review*) | 511282 |
|  | #4 | #1 AND #2 AND #3 | 23 |
| **Ebase** | | | |
|  | #1 | 'sudden deafness'/exp | 3431 |
|  | #2 | 'deafness, sudden':ab,ti OR 'hearing loss, sudden':ab,ti OR 'sudden hearing loss':ab,ti OR 'sudden  deafness':ab,ti | 7485 |
|  | #3 | #1 OR #2 | 7485 |
|  | #4 | 'hyperbaric oxygen therapy'/exp | 21461 |
|  | #5 | 'hbo-therapy':ab,ti OR 'high pressure oxygen':ab,ti OR 'high tension o2':ab,ti OR'high tension  oxygen':ab,ti OR 'hyperbaric medicine':ab,ti OR 'hyperbaric o2':ab,ti OR 'hyperbaric oxygen'  :ab,ti OR 'hyperbaric oxygen treatment':ab,ti OR 'hyperbaric oxygenation':ab,ti OR 'hyperbaric  oxygenization':ab,ti OR 'hyperbaric therapy':ab,ti OR 'oxygen, hyperbaric':ab,ti OR 'hyperbaric  oxygen therapy':ab,ti | 14969 |
|  | #6 | #4 AND #5 | 23179 |
|  | #7 | 'systematic review'/exp OR 'review, systematic':ab,ti OR 'systematic review':ab,ti | 557704 |
|  | #8 | 'meta analysis'/exp OR 'meta analysis':ab,ti OR 'meta analyses':ab,ti | 423300 |
|  | #9 | #7 OR #8 | 719079 |
|  | #10 | #3 AND #6 AND #9 | 28 |
| **Scopus** | | | |
|  | #1 | TITLE-ABS-KEY (("sudden sensorineural hearing loss") OR ("sudden deafness") OR ("Sudden  Hearing Loss") OR ("Deafness, Sudden") OR ("Sudden Deafness")) | 6431 |
|  | #2 | TITLE-ABS-KEY(("Hyperbaric Oxygenation") OR ("Hyperbaric Oxygenations") OR("Oxygenations, Hyperbaric") OR ("Hyperbaric Oxygen Therapy") OR ("Oxygen Therapies, Hyperbaric") OR  ("Oxygen Therapy, Hyperbaric") OR ("Therapies, Hyperbaric Oxygen") OR ("Therapy, Hyperbaric Oxygen") OR ("Oxygenation, Hyperbaric")) | 17312 |
|  | #3 | ("Meta-Analysis as Topic" OR "Meta-Analy*" OR "Systematic Reviews as Topic" OR  "Systematic Review*") | 724646 |
|  | #4 | #1 AND #2 AND #3 | 27 |

1. **The list of excluded articles by full text screening with exclusion reason**

| van der Veen EL,et al.2014 | It’s not related to sudden sensorineural hearing loss |
| --- | --- |
| Chaushu H,et al.2023 | It’s not related to efficacy of hyperbaric oxygen |

1.van der Veen EL, van Hulst RA, de Ru JA. Hyperbaric Oxygen Therapy in Acute Acoustic Trauma: A Rapid Systematic Review. *Otolaryngol Head Neck Surg*. 2014 Jul;**151**:42-5. doi: 10.1177/0194599814526555. Epub 2014 Mar 19. PMID: 24647641.

2.Chaushu H, Ungar OJ, Abu Eta R, Handzel O, Muhanna N, Oron Y. Spontaneous recovery rate of idiopathic sudden sensorineural hearing loss: A systematic review and meta-analysis. *Clin Otolaryngol*. 2023 May;**48**:395-402. doi: 10.1111/coa.14036. Epub 2023 Jan 22. PMID: 36640119.
